# Assessing the Awareness on Symptoms and Risk Factors of Stroke amongst Rural Community in Central Region of Malaysia: A Cross-Sectional Survey

**DOI:** 10.21315/mjms2024.31.1.13

**Published:** 2024-02-28

**Authors:** Nik Nasihah Nik Ramli, Deviga Genasan, Norzawani Shafika Rossman

**Affiliations:** 1 School of Graduate Studies, Management and Science University, Selangor, Malaysia; 2 International Medical School, Management and Science University, Selangor, Malaysia

**Keywords:** stroke, signs and symptoms, risk factors, awareness, rural population

## Abstract

**Background:**

As the third leading cause of mortality in Malaysia, stroke is recognised as a medical emergency which requires urgent medical attention within a limited timeframe to prevent exacerbation of the brain damage and death in patients. Recent report revealed a high prevalence of pre-hospital delay amongst the stroke patients due to the lack of awareness on symptoms and risk factors of stroke, as well as poor understanding on appropriate action towards stroke. A number of studies had assessed stroke awareness amongst urban population residing in central region of Malaysia but yet amongst rural population.

**Methods:**

A cross-sectional survey was conducted amongst individuals residing in rural districts of Selangor by using a set of questionnaires assessing the sociodemographic characteristics, as well as the awareness and action towards stroke symptoms and risk factors.

**Results:**

All 343 respondents were able to recognise at least one modifiable risk factor for stroke. Meanwhile, only 36.44% were able to identify all the stroke symptoms. Despite majority of them were familiar with the stroke term, less than half of the respondents were aware of calling the emergency medical service as the appropriate action towards stroke symptoms.

**Conclusion:**

The present study indicated a poor level of awareness and action towards stroke symptoms and risk factors amongst rural population residing in Selangor.

## Introduction

Cerebrovascular accident or stroke occurs when the blood circulation supplying to the brain is blocked (ischaemic stroke) or ruptured (haemorrhagic stroke) causing the affected brain area to become damaged, leading to functional disability or even death (1). There are several factors associated with the high risk of cerebrovascular accident in individuals. The non-modifiable stroke risk factors include age, ethnicity, family history and gender, whereas the modifiable stroke risk factors are hypertension, smoking habit, waist-to-hip ratio, unhealthy diet, sedentary behaviour, dyslipidemia, diabetes mellitus, alcohol consumption, atrial cardiopathy and apolipoprotein B to A1 ratio (2). When an individual suffered a stroke attack, the common spontaneous symptoms include sudden paralysis or weakness of arm, leg or the face on one side of the body leading to difficulty in swallowing, walking and loss of body coordination. Moreover, difficulty to speak, comprehending speech, visual problems in one or both eyes and sudden severe headaches are also amongst the alarming signs of stroke (1).

Around 87% of the total cases of stroke has been recorded as the ischaemic type (3). The focus of acute ischaemic stroke treatment and management is to maintain tissue area of oligemia by immediately restoring blood flow to the affected brain areas and enhancing collateral blood flow by using recombinant tissue plasminogen activator (rt-PA) that functionally breaks up the blood clot, and thus, allowing the blood to reach brain (4). This treatment, however, is only applicable within 4.5 h of stroke onset, whereas administration of rt-PA beyond 4.5 h was reported to be associated with deleterious side effects, notably, hemorrhagic transformation which could lead to high mortality in stroke patients (5). Therefore, lack of awareness on symptoms, risk factors as well as action towards stroke would result in pre-hospital delay and disqualify the patients for effective reperfusion therapy.

Previous reports on awareness of stroke symptoms and risk factors were mostly conducted amongst urban population of Malaysia. In 2014, a similar survey conducted amongst residents in Kajang reported that only 35% of the respondents had satisfactory knowledge of the stroke warning signs, while 29% had satisfactory knowledge on the stroke risk factors. Although 78% can correctly recall emergency medical systems (EMS) number, only 11% were aware of calling the ambulance as the most appropriate action towards witnessing stroke symptoms in individuals (6). On the other hand, a more recent study conducted amongst Petaling Jaya residents in 2019 reported that 82.9%–92.1% of the respondents were able to recognise common symptoms of stroke followed by 74.2% of the study respondents saying that they would go to hospital within 4.5h of stroke onset (7). Studies on stroke awareness amongst rural population is still scarce, therefore, the present study aimed to explore the level of awareness and action towards stroke symptoms and risk factors amongst rural population in Selangor, Malaysia.

## Methods

### Study Design and Setting

This study employed a cross-sectional questionnaire-based research conducted amongst adult population from the rural areas in Selangor. The participants were sampled from Hulu Selangor, Sabak Bernam and Kuala Langat districts based on the list of zones recognised as rural areas by the Selangor state council (8). Necessary ethical approval for this study was obtained from the University Ethics Committee of Management and Science.

### Study Size

The sample size was determined and calculated by using Cochrane’s sample size formula based on the prevalence of a previous study on the level of colorectal cancer awareness amongst the rural population in Malaysia (9). Thus, the measured sample size required was 326.

### Participants

The participants were sampled from individuals residing in Hulu Selangor, Sabak Bernam and Kuala Langat districts which are recognised as rural areas by the Selangor state council. The eligibility criteria included those individuals aged 18-year-old and above who currently residing in the aforementioned rural areas. Non-probability snowball sampling was used to recruit the participants for this study, whereby the survey questionnaire was disseminated by the village heads (*ketua kampung*) to their respective community. The participants received a one-time survey after obtaining their informed consent.

### Data Measurement

This study used a set of validated questionnaires developed by Ahmed et al. (10) and the bahasa Malaysia version of the questionnaire was generated as per the guideline’s recommendation. A pilot study conducted by using 40 respondents demonstrated an overall Cronbach’s alpha value of 0.877, indicating a very strong level of reliability of the instrument. The Bahasa Malaysia version of the questionnaire were distributed to the rural communities as it is the national language which can be comprehended by majority of Malaysians regardless of their sociodemographic background. The questionnaire consisted of two sections: the first section assessed the sociodemographic characteristics of the respondents, while the second section assessed the awareness and action towards stroke signs, symptoms and risk factors.

### Statistical Analysis

The data were analysed by using a descriptive analysis with Statistical Package for the Social Sciences software (IBM Corp., SPSS version 26.0, Armonk USA). The categorical and descriptive data were cross tabulated to obtain frequency and percentages. Shapiro-Wilk normality assumption test for all data did not achieve the normality assumption (*P* < 0.001) and therefore the correlation between demographic characteristics and the awareness of stroke symptoms and risk factors were assessed by using Spearman’s rho correlation analysis. Based on the correlation coefficient (*r_s_*), the association strength was interpreted as small (*r_s_*: 0.1–0.3), moderate (*r_s_*: 0.3–0.5) or strong (*r_s_*: > 0.5). Multiple linear regressions utilising the enter model were used to estimate the influence of the demographic factors on the associations. Value of *P* < 0.05 was recognised to be statistically significant.

## Results

### Sociodemographic

Based on Table 1, there were 176 (51.3%) females and 167 (48.7%) males who had participated in this study. Majority of the respondents were Malays (97.1%) from the age group of 26 years old to 35 years old (27.4%), with married status (69.4%), graduated from university (49.6%), currently employed (67.9%) and acquired less than RM2,000 of monthly income (45.2%). There were only 14% (48) of the respondents who reported to have smoking habit, while the rest of them were non-smokers. In terms of cardiovascular health risk, 67 (19.6%) of the respondents reported to suffer from hypertension, 42 (12.3%) with hypercholesterolemia, 22 (6.4%) with diabetes, 11 (3.2%) with heart disease, 6 (1.8%) had earlier cerebral stroke, while 29 (8.5%) reported to have other diseases. Majority of the respondents had heard about stroke (96.2%), while there were 258 (75.4%) respondents who reported to have acquainted with a stroke patient.

### Awareness of Stroke Risk Factor

All respondents were able to identify at least one modifiable risk factors for stroke. On average, the mean score was 8.9, ranging from 0 to 13 (64.5%). The most recognised stroke risk factor was hypertension (88.3%) followed by stress (81.6%), hypercholesterolemia (81%), unhealthy diet (77.3%), heart disease (76.1%), lack of exercise (71.1%), family history (70.8%), smoking (68.8%), obesity (67.3%), atrial fibrillation (66.8%), alcohol consumption (63.3%) and diabetes (63.0%). Meanwhile, 80 (23.3%) respondents mistakenly identified cough as the stroke risk factor for stroke (Table 2).

### Awareness of Stroke Symptoms

Thirty-six percent of the respondents were able to identify all five symptoms of stroke. On average, the mean score was 3.9, ranging from 0 to 8 (48.8%). The most recognised stroke symptom was sudden numbness or weakness of the face, arm or leg (86%) followed by sudden trouble walking, dizziness, loss of balance or coordination (77.8%), sudden confusion, trouble speaking or understanding speech (69.7%), sudden trouble seeing in one or both eyes (62.4%) and sudden severe headache with no known causes (55.1%). There were respondents who misidentified sudden nosebleed (19.5%), sudden vomiting (11.4%) and high body temperature (8.7%) as stroke symptoms (Table 3).

### Action towards Stroke Symptoms

Based on Table 4, most of the respondents (96.2%) acknowledged that stroke is an emergency condition. However, upon asking their action towards stroke symptoms, majority of the respondents (60.9%) answered that they will bring the stroke patient to hospital or clinic, while only 109 (31.8%) respondents answered the correct action which is calling the ambulance. The remaining respondents answered to give aspirin (3.5%), calling the healthcare provider (2.62%), or call the family members of the stroke patient (1.2%).

### Correlation and Regression Analysis

Spearman’s rho correlation analysis showed a small to medium strength of association between the mean awareness on stroke symptoms or the mean awareness on stroke risk factors with the demographic characteristics. There were significant positive correlations between the level of income and the awareness of stroke symptoms (*r_s_* = 0.113, *P* = 0.036), as well as the awareness of stroke risk factor (*r_s_*= 0.117, *P* = 0.031). Analysis of multiple linear regression summarised in Table 5 further showed that respectively, smoking habit and acquaintance with a stroke patient significantly influenced the awareness on stroke risk factors (*P* = 0.018, *P* < 0.001), awareness on stroke symptoms (*P* = 0.001, *P* < 0.001), as well as action towards stroke symptoms (*P* = 0.027, *P* = 0.009).

## Discussion

### Awareness on Stroke Risk Factors

The present study reported a moderate level of awareness on stroke risk factors amongst the rural communities in Selangor. Similar levels of awareness on stroke risk factors were observed amongst general population in urban areas of Pahang and Federal Territories states (6, 11). Additionally, majority of the stroke patients admitted to general hospital in Kelantan were identified to have lack of knowledge and awareness on stroke (12). According to the 2015 Nationwide Health and Morbidity Survey (NHMS), the national prevalence of hypertension amongst Malaysian adults was 30.3% (13). The urbanisation phenomenon, sedentary lifestyle and high salt as well as fatty food consumption may have contributed to the rising prevalence of hypertension (14). In contrast, the prevalence of hypertension amongst the rural population in Selangor is relatively low (19.6%). This finding was supported by another study, which discovered that the prevalence of hypertension was found to be lower amongst Asian people living in rural areas, whereby the frequency of hypertension is two to three times lower as compared to individuals living in urban areas (15).

Interestingly, despite the prevalence of diabetes around the globe, studies in Korea and Portugal have reported that diabetes was a poorly recognised stroke risk factor (16, 17). Pancioli et al. (18) previously observed the lack of understanding on how diabetes leads to stroke, with just 13% of the diabetic patients in Ohio were able to identify diabetes as a major stroke risk factor. Likewise in this study, diabetes was also the least recognised stroke risk factor amongst the rural population in Selangor. Based on National Diabetes Registry, there were 902,991 active diabetes patients at the end of the reporting year of 2020, whereby nearly 99% of these diabetic patients had type 2 diabetes (19). Furthermore, diabetes is expected to affect 7 million Malaysian adults aged 18 years old and above by the year 2025 (15). This highlights the importance and urgency of conducting health awareness campaigns as Malaysia is notoriously known as the most diabetic country amongst Southeast Asia countries (20).

### Awareness and Action towards Stroke Symptoms

The awareness on the stroke symptoms amongst rural population in Selangor was considered poor as only 36.44% were able to identify all five symptoms of stroke. A similar study conducted in Kajang which is an urban district of Selangor also reported that only 35% of the respondents had satisfactory knowledge of the stroke warning signs (6). On the other hand, a more recent study conducted amongst Petaling Jaya residents in 2019 reported that 82.9%–92.1% of the respondents were able to recognise the common symptoms of stroke (7). In contrast, Ching et al. (7) study conducted at 42 health screening centres in Malaysia reported 74.3% of the respondents were able to identify all the symptoms of stroke (7).

Previous studies conducted amongst urban community of Singapore and the United States by the Centres for Disease Control and Prevention (CDC) in 2001 reported that the most acknowledged stroke symptom was sudden numbness or weakness on one side of the body with 92.7% of respondents answered correctly (21, 22). Similar to the current study amongst rural population in Selangor, hemiparesis was the most recognised stroke symptoms with 86% of respondents answered correctly. However, this is in contrast with a nationwide study by Ching et al. (7), whereby the most commonly identified symptom was slurred speech which was answered correctly by 92.1% of the total respondents (7).

In the present study, the least recognised symptom of stroke is the sudden severe headache with no known causes. This is possibly due to their exposure from the public education campaign of ‘Face, Arm, Speech, Time (FAST)’ which is the acronym commonly used to promote awareness on stroke warning sign. Each letter in FAST indicates the sign of stroke such as F-face drooping, A-arm weakness, S-speech and T-time. The Malaysian Stroke Council (MSC) and Boehringer Ingelheim’s Angels Initiative have collaborated to provide the FAST Heroes initiative to schools and kindergartens across Malaysia (23). The campaign aims to raise awareness of stroke symptoms amongst youngsters. Bidin et al. (24) assessed a pre- and post-questionnaire on stroke knowledge, action and attitude amongst individuals who attended media-driven health promotion efforts, such as the ResQ Stroke Campaign (RSC) in connection with World Stroke Month 2021 in Malaysia. The findings showed a considerable improvement in the public’s capacity to recognise stroke warning signals, particularly those highlighted by the FAST campaign, as well as a corresponding decrease in those who held incomplete or incorrect ideas about stroke (24). Since headache is not included in FAST, hence this may be the reason headache is the least recognised risk factor in this study.

### Action to Stroke Symptom

Approximately 60% of the respondents deemed that taking the stroke patient to a hospital or clinic is the appropriate action. This may result from their perception that delivering the stroke patients to the hospital themselves is faster than waiting for the ambulance arrival. Prior community studies of patients with acute stroke, conducted by Williams et al. found that patients who utilised the 911 emergency telephone system arrived at the hospital substantially sooner than patients who did not use the 911 system (25, 26). The study found that individuals who had previously experienced a stroke or were aware of stroke warning signs did not appear earlier than those who were unaware of stroke symptoms. Whereas, the factor that was associated with early presentation was the timing of ambulance arrival (26).

It is important to note that ambulance is a medically equipped vehicle along with the presence of trained medical personnel. Rapidly expanding over the world, the concept of mobile stroke unit which is specialised ambulances equipped with a CT scanner, allows brain imaging to be conducted at the patient’s location, hence bringing the ‘hospital to the patient’ (27, 28). A mobile stroke unit is equipped with both the medical competence and imaging technology that allows a fast detection of the type of stroke in order to proceed with intravenous tissue plasminogen activator if suitable. Another advantage is that the neurology consultation takes place at the meeting location. If a patient does not require tertiary care, then they can be returned to the local hospital, eliminating unnecessary transfers to emergency rooms (29, 30). These units may also be implemented in rural and distant regions to improve the quality of treatment delivered to individuals suffering from an acute stroke (30). A study by Shuaib et al. (31) found that mobile stroke unit provides an innovative technique to evaluating and treating patients with suspected strokes in rural areas, potentially reducing admissions to overcrowded tertiary care centres. Therefore, understanding this knowledge is essential in creating effective public education programmes for the rural population.

### Correlation Study

The awareness on stroke symptoms and risk factors amongst the respondents were found to be significantly correlated with their income level. A study conducted amongst Ugandan rural community in 2015 reported that there was low level of awareness on stroke amongst individuals with a lower income and education level (32). This may be due to higher income individuals having more access and demand to the healthcare system (11). A study in China by Yuan et al. (33) found that the greatest barrier to contacting EMS in East, South and Central regions of China was associated with the lack of awareness on action towards stroke symptoms, while the main barrier to calling EMS amongst North and Northeast communities was associated with low-income level. The occurrence of regional differences in stroke awareness and poverty necessitates the development of specialised stroke education initiatives for the targeted regions and populations in China (33). Similarly, studies conducted amongst populations of New Zealand, Jordan and the United States also discovered that lower education and income significantly correlate with lower awareness on stroke (33–35).

Interestingly, several studies had reported that non-smokers were better informed on the stroke than those with smoking habit. Relevant study from Vietnam reported that the urban community were more knowledgeable on the harmful effects of smoking compared to those living in rural areas (36). Furthermore, those with smoking habit displayed significant lower knowledge with only 59.4% correctly identified smoking as risk factor for stroke as compared to the non-smokers (70.3%) (36). A similar study from Israel also reported only 21.6% of smokers gave correct answers as compared to the non-smokers (50%) when they were asked about the consequence of smoking on human lifespan, prolonged exposure to second-hand smoke on lung and heart, as well as correlation of smoking with general health problems (37). Clearly, smokers acknowledged the health hazards of smoking to a certain extent, yet they tend to underestimate the risk on themselves (38). Evidently, a recent meta-analysis study revealed that pictorial cigarette pack warnings significantly impacted the motivations towards smoking cessation amongst smokers but not their beliefs about their own health risks (39).

Closer interpersonal and interfamily relationships were also shown to be associated with a higher level of familiarity with stroke which emphasises the need of education to the general population about stroke signs and risk factors (18). A cross-sectional questionnaire study of 11 German stroke support groups revealed that they had strong knowledge on stroke, which might be attributed to their expertise and personal experience with their family member who had stroke (40). This showed that individuals who heard or known a stroke patient have a better awareness and knowledge regarding stroke. A cross-sectional study done at the neurology clinic and polyclinic of a university hospital in Izmir, Turkey reported that primary caregivers of stroke patients have higher level of awareness on stroke than caregivers of non-stroke patients (40). Although, Saad et al. (41) highlighted that education level of stroke patients’ caregivers does play a significant role affecting their awareness level.

## Conclusion

The awareness of stroke symptoms, risk factors and appropriate action towards stroke was found to be poor amongst the rural communities in Selangor, Malaysia which shown to be impacted by their level of income, smoking status, and acquaintance with stroke patients. Thus, it is suggested that future intervention programmes should focus on effective health promotion programmes and regular community health screening for stroke risk factors in order to promote the awareness, hence mitigating the mortality and morbidity associated with cerebrovascular diseases.

## Figures and Tables

**Table 1:** Sociodemography characteristics of the respondents

Characteristic		*n*	%
Gender	Male	167	48.7
	Female	176	51.3
Age (years old)	18–25	53	15.5
	26–35	94	27.4
	36–45	85	24.8
	46–55	74	21.6
	56–64	28	8.2
	More than 64	9	2.6
Social status	Single	82	23.9
	Married	238	69.4
	Divorced	13	3.8
	Widowed	10	2.9
Education	Primary education	5	1.5
	Secondary education	152	44.3
	Diploma or certificates	16	4.7
	University	170	49.6
Employment	Studying	21	6.1
	Unemployed	89	25.9
	Employed	233	67.9
Ethnicity	Malay	333	97.1
	Chinese	5	1.5
	Indian	4	1.2
	Others	1	0.3
Income	Less than RM2,000	155	45.2
	RM2,000–RM3,999	104	30.3
	RM4,000–RM6,000	66	19.2
	More than RM6,000	18	5.2
Comorbidities	Hypertension	57	16.6
	Diabetes	32	9.3
	Hypercholesterolemia	42	12.2
	Heart disease	11	3.2
	Stroke	6	1.7
	Smoking	48	14.0
	Other diseases	30	8.7
Heard of stroke	No	13	3.8
	Yes	330	96.2
Know a stroke patient	No	84	24.5
	Yes	259	75.5

**Table 2:** Frequency and percentage of correct answers on stroke risk factors

Stroke risk factors	*n*	%
Hypertension	303	88.3
Stress	280	81.6
Hypercholesterolemia	278	81.0
Unhealthy diet	265	77.3
Heart disease	261	76.1
Lack of exercise	244	71.1
Family history	243	70.8
Smoking	236	68.8
Obesity or Overweight	231	67.3
Atrial fibrillation	229	66.8
Alcohol consumption	217	63.3
Diabetes	216	63
Cough (trap question)	80	23.3

**Table 3:** Frequency and percentage of correct answers on stroke symptoms

Stroke symptoms	*n*	%
Sudden numbness or weakness of face, arm or leg	295	86.0
Sudden trouble walking, dizziness, loss of balance or coordination	267	77.8
Sudden confusion, trouble speaking or understanding speech	239	69.7
Sudden trouble seeing in one or both eyes	214	62.4
Sudden severe headache with no known cause	189	55.1
Sudden nosebleed (trap question)	67	19.5
Sudden vomiting (trap question)	39	11.4
High temperature (trap question)	30	8.7

**Table 4:** Action to stroke symptoms

Items	*n*	%
Do you think stroke require a prompt treatment?		
Yes	330	96.2
No	13	3.8
If someone shows sign and symptoms of stroke, what do you think you should do first?		
Bring to hospital or clinic	209	60.9
Call the ambulance	109	31.8
Give aspirin	12	3.5
Call the healthcare provider	9	2.6
Call the family member	4	1.2

**Table 5:** Multiple linear regression analysis identifying demographic factors associated with awareness on stroke risk factors, symptoms and action towards stroke

Demographic characteristics	Awareness on stroke risk factors		Awareness on stroke symptoms		Action towards stroke symptoms	
		
	*β*-coefficient	*P-*value	*β*-coefficient	*P-*value	*β*-coefficient	*P-*value
Gender	−0.279	0.181	−0.174	0.679	0.051	0.553
Age	0.006	0.537	0.019	0.313	0.005	0.257
Social status	−0.253	0.154	−0.308	0.390	−0.168	**0.023**
Education	−0.006	0.950	0.176	0.388	−0.008	0.843
Employment	−0.295	0.081	−0.007	0.982	−0.006	0.928
Ethnicity	−0.615	**0.049**	−0.381	0.545	0.017	0.896
Income	0.175	0.159	0.049	0.844	0.068	0.189
Comorbidities	−0.102	0.162	−0.211	0.153	−0.024	0.422
Smoking habit	−0.679	**0.018**	−1.963	**0.001**	−0.264	**0.027**
Know a stroke patient	1.447	**< 0.001**	2.919	**< 0.001**	−0.246	**0.009**
